# The extracts of *Astragalus membranaceus* overcome tumor immune tolerance by inhibition of tumor programmed cell death protein ligand-1 expression

**DOI:** 10.7150/ijms.42978

**Published:** 2020-03-26

**Authors:** Hsu-Liang Chang, Yi-Hsuan Kuo, Li-Hsien Wu, Chih-Min Chang, Kai-Jen Cheng, Yu-Chang Tyan, Che-Hsin Lee

**Affiliations:** 1 Department of Internal Medicine, Kaohsiung Municipal Ta-Tung Hospital, Kaohsiung Medical University Hospital, Kaohsiung Medical University, Kaohsiung 80145, Taiwan; 2 Department of Biological Sciences, National Sun Yat-sen University, Kaohsiung 80424, Taiwan; 3 Division of Metabolism, Department of Internal Medicine, Chang Gung Memorial Hospital, Kaohsiung 833, Taiwan; 4 Division of Nephrology, Department of Internal Medicine, Kaohsiung Municipal United Hospital, Kaohsiung 80457, Taiwan; 5 Department of Medical Imaging and Radiological Sciences, Kaohsiung Medical University, Kaohsiung 80145, Taiwan; 6 Department of Medical Research, China Medical University Hospital, China Medical University, Taichung 40402, Taiwan; 7 Department of Medical Laboratory Science and Biotechnology, Kaohsiung Medical University, Kaohsiung 804, Taiwan; 8 Doctoral Degree Program in Marine Biotechnology, National Sun Yat-sen University, Kaohsiung 80424, Taiwan; 9 Aerosol Science Research Center, National Sun Yat-sen University, Kaohsiung, Taiwan, 80424, Taiwan

**Keywords:** the extracts of *Astragalus membranaceus* (PG2), programmed cell death protein ligand-1, tumor immune tolerance

## Abstract

A polysaccharide isolated from the radix of *Astragalus membranaceus*, called PG2, used in traditional Chinese medicine, with potential hematopoiesis inducing and immunomodulation activities. PG2 extracted from *A. membranaceus* has been demonstrated as a novel alternative medicine for cancer patients. Recently, we demonstrated that PG2 enhanced chemotherapy through bystander effect and reduced the expression of indoleamine 2, 3-dioxygenase 1 in tumor cells. Many tumors have been proven to have a high expression of programmed cell death protein ligand-1 (PD-L1), which binds with programmed cell death protein-1(PD-1) in immune cells, thus causing immune tolerance within the tumor microenvironment. With decreased expression of PD-L1, increased immune response can be observed, which might be helpful when developing tumor immunotherapy. The antitumor therapeutic effect mediated by PG2 may associate with an inflammatory immune response at the tumor site. However, the molecular mechanism that by which PG2 inhibits PD-L1 is still incompletely known. The expression of PD-L1 was decreased after tumor cells were treated with PG2. In addition, the cell signaling pathway in tumor cells was evaluated by Western blotting analysis after PG2 treatment. PG2 can downregulate the expression of PD-L1 on the cell surface via the protein kinase B (Akt)/mammalian target of rapamycin (mTOR)/ribosomal protein S6 kinase beta-1 (p70S6K) pathway. In conclusion, our results indicate that PG2 inhibits PD-L1 expression and plays a crucial role in immunotherapy, which might be a promising strategy combined with other treatments.

## Introduction

The immune system is a host's defense mechanism to rid-off various health problems from infection to unhealthy cells. Molecular checkpoints, like programmed cell death protein 1(PD-1) and programmed cell death protein ligand 1 (PD-L1), protect the normal cells from being subjected to destruction during an immune response 1. In tumor microenvironment, high-expression of these checkpoints on cancer cell surface leads to an escape from immune cell recognition, thereby, promoting immune tolerance 2.* Astragalus membranaceus* (PG2) is a botanical-derived drug extracted from the root of *A. membranaceus*. The *A. membranaceus* is traditional Chinese medicine, an important aspect of alternative medicine that is widely used in the treatment of inflammatory diseases, tumors, radical scavenger activity, various cardiovascular diseases, and neuroprotective activity 3. For tumor treatment, it is mostly used to decrease the side effect of chemotherapy known as tumor-related fatigue 4. In recent years, PG2 is found to have anti-cancer effects, particularly when combined with chemotherapeutic drug 2. When chemotherapy is used alongside PG2 treatment, the treatment outcome improves significantly based on clinical studies as seen in patient's improved physical fitness 5. Nevertheless, the mechanisms of PG2 in the regulation of host immunity and therapeutic response remain unclear. Previously, PG2 stimulated host immunity by reducing the expression of indoleamine 2, 3-dioxygenase (IDO) 2. Here, we investigated whether PG2 can decrease tumor immune tolerance by modulating immune checkpoints through the suppression of PD-L1 protein expression. We use murine 4T1 breast and murine CT26 colon cancer cells as models. In this study, we established a co-culture system, that is, tumor cells with either murine WEHI-3 leukemia or murine EL4 lymphoblast cells. Our findings further revealed a significant decrease in the inhibitory signal to T cells co-cultured with PG2-treated tumor cells as seen in the activated protein kinase B (Akt)/mammalian target of rapamycin (mTOR) pathway in immune cells. Moreover, the activated T cells may be accounted for by the decreased in cleaved caspase-3 expression which may then elicit tumor-specific immune attack. The tumor animal models were used to determine the efficiency of PG2/cisplatin combination therapy in the reduction of tumor immune tolerance* in vivo*. Based on these data, we provide a new mechanism for PG2 treatment as part of immunotherapy.

## Materials and Methods

### Reagents, cells and mouse

The root of *A. membranaceus* (PG2) purchased from PhytoHealth Corp (Taipei, Taiwan) 6. Cisplatin was purchased from the Sigma-Aldrich. Murine 4T1 and CT26 cells were cultured in Dulbecco's modified Eagle's medium (DMEM) supplemented with 50 μg/ml gentamicin and 10% heat-inactivated fetal bovine serum at 37ºC in 5% CO_2_. Murine WEHI-3 leukemia or murine EL4 lymphoblast cells were cultured in Iscove's Modified Dulbecco's Medium (IMDM) contains 4 mM L-glutamine, 4500 mg/L glucose, and 1500 mg/L sodium bicarbonate and 10% heat-inactivated fetal bovine serum at 37ºC in 5% CO_2_. Constitutively active AKT plasmid was described previously 7. The BABL/c mice were purchased from the National Laboratory Animal Center of Taiwan. The experimental protocol was approved by the Laboratory Animal Care and Use Committee of the National Sun Yat-sen-University (permit number: 10714).

### Cell viability assay

Cells were treated PG2 (0-10000 ng/ml) in serum- free medium for 24 h. The adherent cells were measured for cell survival. Cell proliferation was assessed by Cell Counting Kit-8 (Sigma-Aldrich) according to the manufacturer's instructions 8.

### Western blot analysis

The Bicinchoninic Acid (BCA) protein assay (Pierce Biotechnology, Rockford, IL) was used to determine the protein contents. SDS-PAGE was used to fractionate protein and the fractionated proteins were transferred to nitrocellulose membranes (Pall Life Science, Glen Cove, NY). The antibodies against PD-L1 (GeneTex, Inc. Irvine, CA), phosphorylation-AKT (Santa Cruz Biotechnology Inc, Santa Cruz, CA), AKT (Santa Cruz), phosphorylation- p70s6K (Cell Signaling, Danvers, MA), p70s6K (Cell Signaling), phosphorylation-mTOR (Cell Signaling), mTOR (Cell Signaling), caspase 3 (GeneTex) or β-actin (Sigma-Aldrich, St. Louis, MO) were used to detect targeted protein. Rabbit anti-mouse IgG-peroxidase antibody (Sigma Aldrich) and goat anti-rabbit IgG-peroxidase antibody (Sigma Aldrich) were used as secondary antibodies. Chemiluminescence system (T-Pro Biotechnology, New Taipei City, Taiwan) was used to observe the signals. ImageJ software was used to quantify the signals 9.

### Flow cytometry

For PD-L1 detection, 10^6^ cells were counted and fixed with 70% ethanol in -30°C overnight. Subsequently, PD-L1 antibody (GeneTex, Inc.) was added and stand for 1 h at 4°C and fluorochrome-labeled goat anti-rabbit IgG secondary antibody (GeneTex) for another 30 minutes at 4°C.

### Co-culture system

Tumor cells were plated in 6 well culture plates and treated with PG2 (10,000 ng/ml) for 24 h. The immune cells (murine WEHI-3 leukemia, murine EL4 lymphoblast cells and primary murine T lymphocyte cells) mixed with equal amount of serum-free medium. After 24 h, the immune cells were collected and detected the protein expression for Western blotting.

### Animal study

The subcutaneous 4T1 and CT26 tumor models to evaluate the antitumor efficacy of combination treatment with PG2 and cisplatin. Groups of 5-7 mice that inoculated subcutaneously with 4T1 or CT26 cells (10^6^) at day 0 were intraperitoneal injected with PG2 (50 mg/kg) at day 7, day 9, day 14, day 16, day21, day 23 followed by cisplatin (2 mg/kg) at day 8, 15, and 22, or with either treatment alone. The control mice were treated with PBS. All of the mice were monitored for tumor weight at day 45.

### Statistical analysis

All data were expressed as mean ± standard deviation (SD). The unpaired, two-tailed Student's t test was used to determine differences between groups. Any P value less than 0.05 is regarded statistically significant.

## Results

### PG2 reduced PD-L1 expression *in vitro*

**Figure 1:**
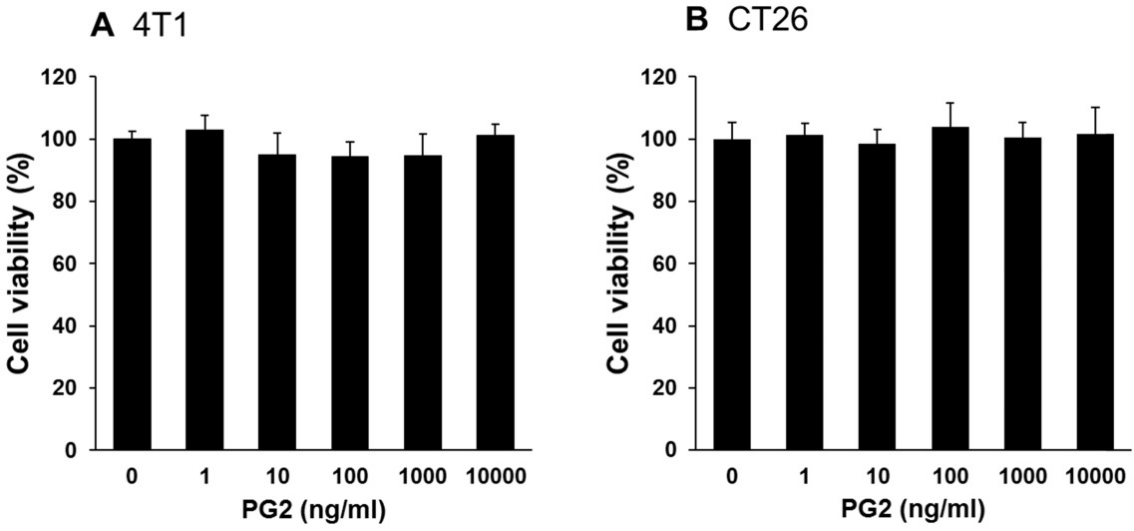
** Effects of PG2 on cell viability in 4T1 and CT26 cells.** (A) 4T1 and (B) CT26 cells were treated with indicated concentrations of PG2 for 24 h. Cell viability was measured by Cell Counting Kit-8 assay. (mean ± SD, n = 6)

**Figure 2:**
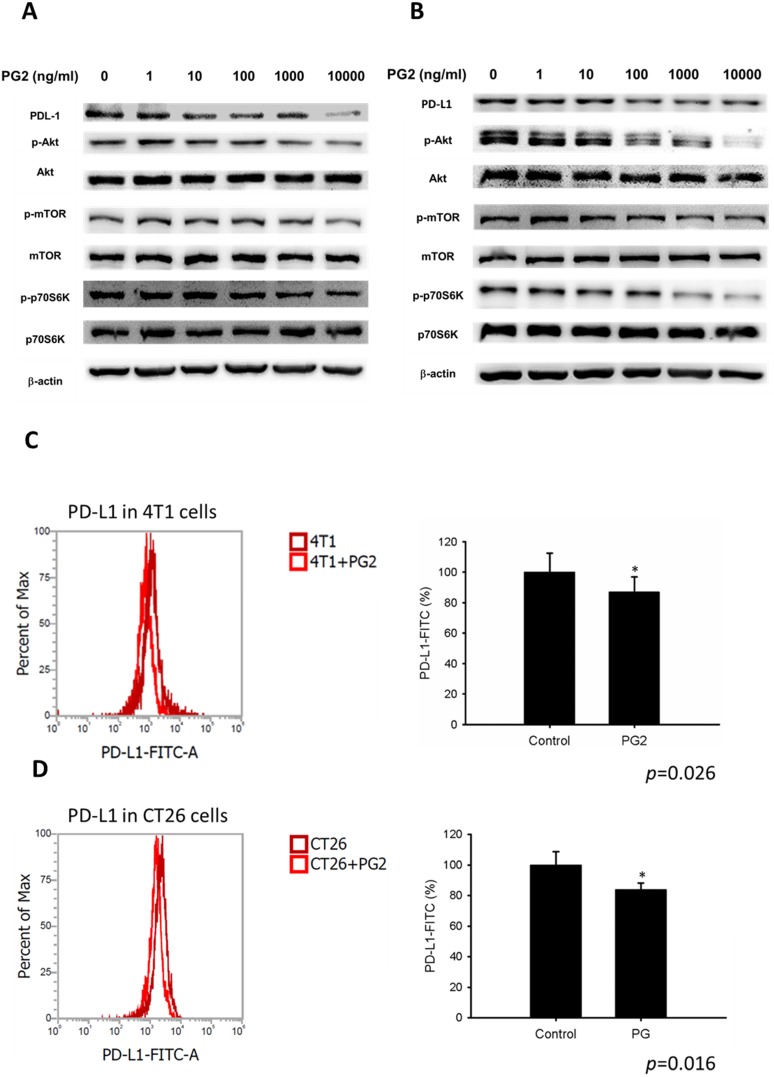
** PG2-mediated PD-L1 protein expression.** PG2 reduced PD-L1 protein expression in (A) 4T1 and (B) CT26 cells in a dose-dependent manner. After treatment with PG2 (0-10 μg/ml) for 24 h, the expression of PD-L1 levels in 4T1 and CT26 cells were measured by Western blotting. PG2 reduced PD-L1 expression through AKT/mTOR/p70S6K signal pathways. PG2 reduced AKT/mTOR/p70S6K signaling pathways in (A) 4T1 and (B) CT26 cells in a dose-dependent manner. After treatment with PG2 (0-10 μg/ml) for 24 h, the expression of AKT/mTOR/p70S6K signaling pathways in 4T1 and CT26 cells were measured by Western blotting. Each experiment was repeated three times with similar results. PG2 reduced PD-L1 expression on tumor surface. PG2 reduced PD-L1 expression on the surface of (C) 4T1 and (D) CT26 cells. After treatment with PG2 (0-10 μg/ml) for 24 h, the expression of PD-L1 on the surface of (C) 4T1 and (D) CT26 cells were measured by flow cytometry. *, p<0.05; ***, p<0.001

Herein, mouse breast cancer 4T1 and colorectal cancer CT26 cells were used to investigate the anti-tumor immunity tolerance activity of PG2. The cell survival, and the expression of PD-L1 after tumor cells treated with various concentrations PG2 are shown in Figure 1 and Figure 2. The different concentrations (0-10,000 ng/ml) of PG2 treatment without significant cytotoxicity after treated for 24 h and used to evaluate the effects of PG2 on PD-L1 production (Figure 1 A and B). The expressions of PD-L1 in 4T1 and CT26 cells were significantly reduced in tumor cells after the treatment of PG2 (Figure 2 A and B). The increased treatment of PG2 significantly downregulated the expression of PD-L1 in two tumor cells. These results demonstrated that PG2 did not influence the cell proliferation but reduce the expression of PD-L1 in tumor cells. PD-L1 expression is associated with various signaling pathway 10. The above findings prompted us to further explore the detailed mechanism underlying the PD-L1 expression of PG2 in tumor cells. The AKT/mTOR/p70S6K signaling pathway involved in the regulation of PD-L1 expression 11. Previously, PG2 inhibited another immune checkpoint protein (indoleamine 2, 3-dioxygenase) expression via AKT/mTOR signaling pathway 2. As shown in Figure 2 A and B, we next examined the AKT/mTOR/p70S6K signaling pathway in PG2-reduced PD-L1 expression. The treatment of PG2 decreased the phosphorylation of AKT, mTOR and p70S6K, indicating down-regulation of the AKT/mTOR/p70S6K pathway by PG2 treatment in 4T1 cells (Figure 2A). Meanwhile, the similar results were observed when PG2 treated with CT26 cells (Figure 2B). Furthermore, the expression of PD-L1 was significantly reduced on the cell surface after the tumor cells were treated with PG2 (10,000 ng/ml) as demonstrated by flow cytometry analysis (Figure 2 C and D). Taken together, these results indicated that reduction of PD-L1 by PG2 in tumor cells was associated with downregulation AKT/mTOR/p70S6K pathway.

### PG2 reduced PD-L1 expression via downregulation AKT signaling pathway

**Figure 3:**
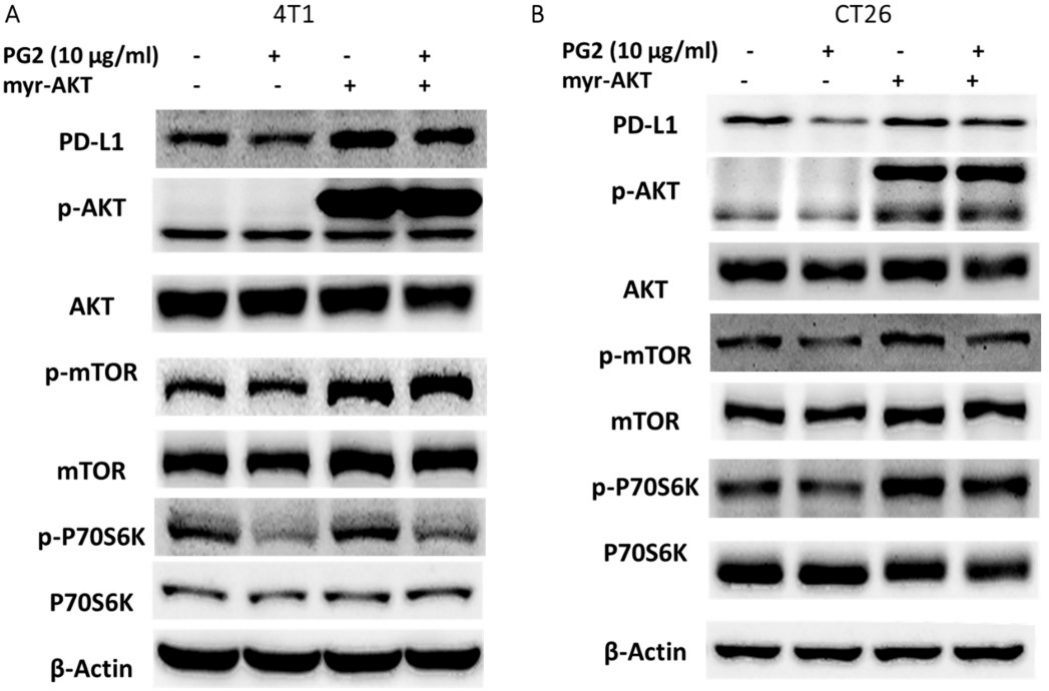
** PG2 reduces PD-L1 expression through AKT pathway.** The (A) 4T1 and (B) CT26 (10^5^) cells were transfected with constitutively active AKT plasmid (5μg) for 16 h prior to treated with PG2 (10µg/ml) for 24 h. The protein expression was measured by Western blotting. Each experiment was repeated three times with similar results.

In this study, we suggested that PG2 reduced the expression of PD-L1 by reducing AKT phosphorylation. By transfecting constitutively active AKT plasmid, the AKT/mTOR/p70S6K signaling pathway will be reversed 12-14. Reductive effects of PG2 on the AKT/mTOR/p70S6K signaling pathway in 4T1 (Figure 3A) and CT26 (Figure 3B) cells were relieved by transfecting constitutively active AKT plasmid. The expression of PD-L1 indeed was increased after transfecting constitutively active AKT plasmid. Transfection of constitutively active AKT plasmid enhanced the expression of PD-L1 after PG2 treatment in comparison with control transfection. Our results point out that downregulation AKT is essential for PG2-reduced PD-L1 expression in 4T1 and CT26 cells. PG2 inhibit tumor PD-L1 expression through modulating the AKT/mTOR signaling pathway.

### PG2-treated cells influenced the protein expression of immune cells

**Figure 4:**
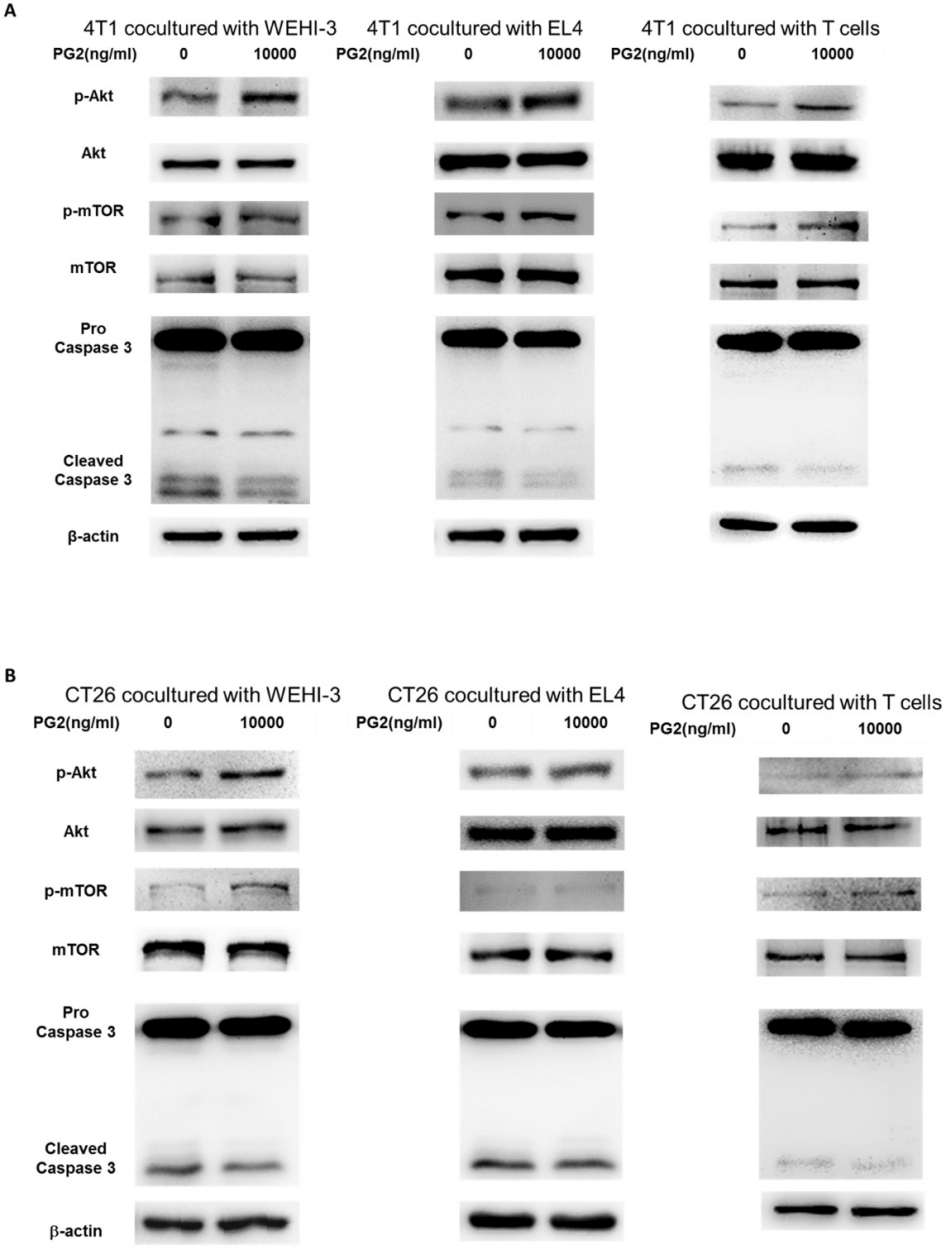
** PG2 affected AKT/mTOR signaling and apoptosis in immune cells.** WEHI-3, EL4 cells and T lymphocytes were co-cultured with PG2 (10μg/ml)-treated (A) 4T1 and (B) CT26 cells prior to harvesting. The protein expression was analyzed by Western blotting. Each experiment was repeated three times with similar results.

The reduction of PD-L1 was correlated with PG2 treatment in the tumor cells. We next investigated whether the reduction of PD-L1 plays a role in PG2-induced immune cell activities after immune cells/ tumor cells co-culture system. The activities of immune cells were rescued after PG2 treatment. Herein, we chose murine WEHI-3 leukemia, murine EL4 lymphoblast cells and primary murine T lymphocyte cells to co-culture with tumor cells treated with PG2. The AKT/mTOR pathway has emerged as a major effector of cell growth and proliferation 15. As shown in Figure 4 A, the AKT/mTOR signal was increased in the WEHI-3, EL4 and primary T lymphocytes cells cultured with 4T1 cells treated with PG2 compared with those treated with PBS. We observed the similar results in murine EL4 lymphoblast cells and primary murine T lymphocyte cells co-cultured with CT26 (Figure 4B). PD‑L1 expressed in tumor cells interacting with its receptor PD‑1 expressed in immune cells could promote T cell apoptosis 16. In Figure 4, the cleaved caspase 3 was significantly reduced in the immune cells cultured with tumor cells treated with PG2. These results reveal that PG2 inhibited the production and function of PD-L1 in tumor cells.

### Combination treatment with cisplatin and PG2 inhibits tumor growth

**Figure 5:**
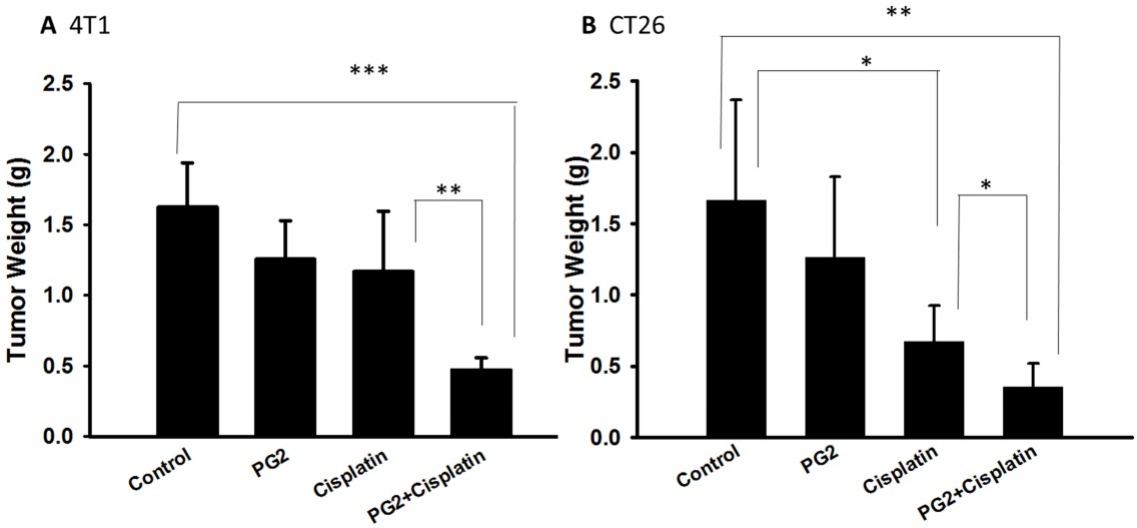
** The antitumor effects of PG2 in combination with cisplatin on tumors.** Groups of 5-7 mice that inoculated subcutaneously with (A) 4T1 or (B) CT26 cells (10^6^) at day 0 were intraperitoneal injected with PG2 (50 mg/kg) at day 7, day 9, day 14, day 16, day21, day 23 followed by cisplatin (2 mg/kg) at day 8, 15, and 22, or with either treatment alone. The control mice were treated with PBS. All of the mice were monitored for tumor weight at day 45. *, p<0.05; **, p<0.01; ***, p<0.001

PG2 did not directly induce tumor cell death (Figure 1). Previously, we found that the combination therapy (cisplatin plus PG2) significant inhibited tumor growth in murine melanoma and lung tumor models 2. Noteworthy, tumor-bearing mice treated with cisplatin shown slightly the inhibition of tumor weight. The combination therapy (cisplatin plus PG2) significant inhibited tumor growth in two tumor models. In this study, the murine breast tumor and colorectal tumor were significantly reduced growth after cisplatin/PG2 therapy (Figure 5).

## Discussion

The PD-1/PD-L1 axis is linked to the suppression and evasion of host immune functions 1. The expression of PD-L1 may be one mechanism for immune evasion used by tumors for promoting effector T cell apoptosis and reducing T cell infiltrating 16. Furthermore, our data demonstrated that PG2 played the role in immune modulation, rather than its direct cytotoxicity on tumor cells. The treatment of PG2 in tumor cells decreased the surface expression and protein level of PD-L1. The combined therapy significantly inhibited tumor growth in tumor-bearing mice. PG2 serves as a critical role of tumor immunotherapy agent.

Here, we investigate the possibility that dysregulation of PD-L1 plays a role in tumor cell driven evasion of immune surveillance. Previously, PG2 not only modulated host immune responses but also enhanced the bystander effect of chemodrugs 2. PG2 could induce gap junction (connexin (Cx) 43) expression. Cx43 increased intercellular tumor antigen transfer between antigen-present cells (dendritic cells or macrophages), thus enhancing tumor-specific T cell activation 17. Herein, we demonstrated that the PG2 enhanced the chemotherapy by stimulating host immunity by reducing the expression of tumor surface PD-L1 expression. The advantages of PG2 are in overcoming tumor immune evasion and enhancing chemotherapy. PG2 has a proven ability to inhibit tumor growth *in vivo*; however, the detailed mechanism for this still requires further investigation. The patients with tumor metastasis injected with PG2 improved their quality of life and reduced the expression of proinflammatory cytokines 18. Meanwhile, PG2 promoted the maturation of dendritic cells and reduced M2 macrophage population in patients with lung cancer 19. Recently, PG2 suppressed tumor growth and metastasis and potentiated cisplatin effect by increasing M1 macrophage activity, reducing angiogenesis, and cancer stem cell population 19. There is interest in looking for their regulation and improving their application, mainly by combination therapies. Because of its strong properties of activating immunity, we believe that PG2 are worth studying and applying to antic immunotherapy.
